# Emergency triage nurses’ perceptions of caring behaviors and the safety of the patient during triage encounters: a grounded theory study

**DOI:** 10.1186/s12912-024-02122-5

**Published:** 2024-07-03

**Authors:** Zvonka Fekonja, Sergej Kmetec, Urška Fekonja, Nataša Mlinar Reljić, Majda Pajnkihar, Matej Strnad

**Affiliations:** 1 Faculty of Health Science, University of Maribor, Žitna ulica 15, Maribor, 2000 Slovenia; 2 Emergency Department, University Medical Centre Maribor, Maribor, Slovenia; 3 Faculty of Medicine, University of Maribor, Maribor, Slovenia; 4 Prehospital Unit, Department for Emergency Medicine, Community Healthcare Center Maribor, Maribor, Slovenia

**Keywords:** Triage, Grounded theory method, Patient safety, Workload, Caring, Perception

## Abstract

**Background:**

Triage is a dynamic process prioritising the patient coming to the emergency department. Caring behaviour and patient safety during the triage process are essential for ensuring a good care experience and treatment outcome.

**Objective:**

To describe triage nurses’ perceptions on caring behaviors and patient safety in the triage area.

**Design:**

Strauss and Corbin’s Grounded theory method was used to develop the model.

**Methods:**

The study was conducted in the emergency department in northeastern Slovenia. Semi-structured interviews were used for data collection, and 19 triage nurses were selected by theoretical sampling, guided by emerging categories between November 2021 and July 2022. The data analysis was conducted according to Strauss and Corbin’s coding framework.

**Results:**

The analysis of the interviews generated one category: The process of creating a caring and safe triage encounter for the patient, together with two categories that explain the key phenomenon: (1) Triage caring and (2) Safety in the triage process. Within the category “Triage caring”, four subcategories were developed: (1) Assurance of triage nurses’ presence, (2) Connectedness, (3) Respectful attitude, and (4) Knowledge and skills. The category Safety in the triage process consists of three identified subcategories: (1) Conception and perception of safety, (2) Factors influencing patient safety, and (3) Improving the triage safety.

**Conclusions:**

The triage nurses’ perceptions about caring for the patient and his safety in the triage area show that caring and safety are inseparably linked and coincide when triaging a patient. Namely, caring for the patient means ensuring the patient’s safety at the same time.

**Implications for the nursing field:**

A better understanding of the importance of triage nurses’ caring behavior and patient safety emerges from the findings, highlighting the challenges faced in a busy emergency department where nurses must balance providing care and responding to patients’ needs while ensuring safety. Findings in the study show that patient care and safety are inseparably linked and coincide when triaging a patient. Moreover, applying caring behaviour during triage encounter results in greater patient safety.

**No patient or public contribution:**

The study’s design, evaluation of the findings, and execution did not need the involvement of patients or the general public. Participants were triage nurses working in the emergency department. Triage nurses were interviewed about their perceptions of triage nurses on caring behaviors and patient safety during triage encounter.

**Supplementary Information:**

The online version contains supplementary material available at 10.1186/s12912-024-02122-5.

## Background

An effective triage system is key to providing an expert and safe level of patient care [1] and is the core of quality care in the emergency department (ED) [2]. Triage represents a complex process [3] designed to ensure that the level of emergency care provided is proportionate to the clinical urgency of the patients [4]. Every patient receives a triage assessment, which is the first key interaction between the patient and the triage nurse (TN). Therefore, when performing this assessment, the TN must effectively and accurately assess the patient’s health status, determine who should be treated first, and allocate medical resources according to the established priority [5]. High professional triage assessment of patient urgency is essential to ensuring patient safety [6].

The triage process is a critical component of patient care, especially in challenging healthcare settings where resources are often limited, and patient volumes can be high [7]. In such environments, the ability to quickly and accurately assess patient acuity is critical to ensure that those with the most urgent needs receive timely care [8]. Triage nurses play a central role in this process [9]. They use their clinical judgement and experience to prioritise patients according to the severity of their condition [3].

In challenging healthcare settings, the demands on triage nurses are exacerbated by factors such as overcrowding, high patient turnover and limited staffing [10]. These conditions can increase the risk of errors and jeopardise patient safety if not managed effectively [11]. Therefore, implementing robust triage systems and deploying sufficient numbers of well-trained triage nurses are essential to ensure a high standard of care [12].

Caring behaviors in triage encompass a range of actions to ensure patient safety, comfort, and well-being and according to Alkahlout and Ahmad [13] involve continuous patient monitoring throughout patients’ ED and hospital stay, effective communication [3], prioritisation of care, emotional support, and coordination with healthcare staff [14]. Triage nurses engage in these behaviors to ensure patient safety and well-being while managing the demands of a busy emergency department [7, 15, 16]. Continuous monitoring involves frequently reassessing patients’ conditions to detect any changes requiring urgent attention [17]. Effective communication ensures that patients and their families are well-informed about the triage process and wait times, which helps reduce anxiety and fosters understanding [3]. Prioritising care based on the severity of conditions allows triage nurses to provide timely and appropriate interventions, thereby improving patient outcomes [2, 3, 7]. Providing comfort measures, such as offering blankets or pain relief, helps to maintain patient comfort during their wait [18]. Emotional support, through empathetic listening and reassurance, is crucial in alleviating patient stress and fostering a supportive environment [3]. Additionally, triage nurses coordinate closely with other medical staff to ensure seamless patient transitions and timely interventions [19]. These behaviors collectively contribute to a safe and supportive triage environment, underscoring the critical role of triage nurses in patient care [13, 20, 21].

The caring behavior of TN is important for patient safety [22], which is the essence of nursing care and is based on humane and interpersonal equal partner relationships [23]. Caring is focused on the patient’s well-being and is delivered when the TN responds to the patient’s needs [24]. A caring encounter by TN must establish a personal caring relationship with the triaged patient and put them at the centre of the treatment [2], including effectiveness, timeliness and equality [25]. The patient must be treated as a human being and as a patient who is aware of his rights and wants to participate in their care and has the opportunity to participate in the decision-making process [24]. Caring is a moral ideal [26] in the ED, ensuring quality, safe and comprehensive care for emergency patients and the satisfaction of patients and healthcare staff [2, 27]. When caring behavior in the ED is evident to patients, they feel comfortable, safe, and trusting that the TN cares for them [28]. Patients who experienced caring behavior in the triage process reported improved emotional and spiritual well-being, a faster sense of improvement in well-being, and a greater sense of safety, comfort and support [29].

While existing research has begun to explore ED-specific safety practices, little is known about the caring behaviors of triage nurses (TNs) and their impact on patient safety during triage encounters. Specifically, there is a notable gap in the literature regarding TNs’ perceptions of caring behaviors and how these perceptions influence patient safety in the triage process. This study addresses this gap by describing TNs’ perceptions of caring behaviors and patient safety in the triage area. Therefore, this study aimed to describe TNs’ perceptions of TN on caring behaviors and patient safety in the triage area. This research aimed to answer the question: What are the TNs’ perceptions about caring behavior and patient safety in the triage encounter?

## Methods

### Study design

We followed a grounded theory design using a Straussian grounded theory (GT) approach [30], as this approach allows the development of substantive theory to explain interpersonal interactions in a particular aspect of human experience [31]. The grounded theory involves an analytical and prescriptive coding framework designed to systematically extrapolate theory from data [32]. The Straussian GT approach [30] was appropriate for several reasons: there is little information on triage nurses’ perceptions of patient safety and caring behavior in an ED; a specific research topic is acceptable; literature reviews can be used to identify knowledge gaps; there are many strategies for analysing structured data; and allows flexibility in integrating researchers’ knowledge and experience into the research process [33].

### Study sample and setting

To investigate the perception of TN on caring and patient safety in the triage area, we conducted semi-structured interviews with 19 TNs working in the ED. None of the participants refused to participate or dropped out of the study. The study was conducted in one of the biggest University hospital in Slovenia. In Slovenia, triage, according to the principles of the Manchester Triage System (MTS), has been chosen as the triage model, which has been demonstrated, according to Slovenian healthcare experts, as the most suitable for implementation in our environment [34]. Moreover, triage is performed by a Registered Nurse who has gained additional specialised knowledge in this area and has at least three years of emergency work experience. The inclusion criteria were as follows: Licenced as a Registered Nurse (RN), RN with at least one year of experience in emergency triage, completed triage course, and direct experience triaging patients in a clinical setting.

The researchers did not establish any formal relationships with the participants before the study began. To recruit participants for this study, we used a two-stage sampling strategy. First, a purposive sample was drawn to identify participants with extensive experience in triage nursing and could provide valuable insights into the research topic [35]. These initial participants were identified through professional networks and emergency department records that could be accessed by the head of the institution. Potential participants were contacted by email or phone. Participants received a participant information sheet containing detailed information about the study’s aims, procedures, possible risks and benefits, the assurance of confidentiality and their right to withdraw from the study at any time without consequences. Participants also had the opportunity to ask questions and seek clarification before giving their informed consent. After conducting the initial interviews, we used theoretical sampling [30] to refine our participant pool further. This approach allowed us to select participants with different characteristics, including gender, age, educational level and work experience, in response to the findings from the initial data analysis. In this way, we were able to ensure a comprehensive exploration of the categories and concepts that emerged over the course of the study.

### Data collection

The first author conducted interviews at the participants’ workplaces at a time convenient to the participants between November 2021 and July 2022. After identifying the eligible and willing subjects, individual interviews were arranged with providers, at this point, informed consent was obtained. All interviews took place at the participants’ workplaces during working hours, either during breaks or at the end of the shift. The dates were set according to the participants’ preferences. The participant and the researcher were only present during the interviews. Interviews lasted 10 to 43 min (M = 17 min, SD = 8.44), and each participant was interviewed once. A semi-structured interview was used to allow interviewees to provide comprehensive and detailed answers without being restricted by overly specific questions. This approach facilitated a more natural flow of conversation and allowed participants to provide rich and in-depth information. We used all questions from the interview guide developed based on a literature review and the first author’s experience, as well as the interview guide tested in the pilot study. Each semi-structured interview began with a brief introduction to the study and obtaining a consent form and permission to audio record the interview. The interviews focused on the TNs’ caring behavior and patient safety during the triage process. The interviews were based on an interview guide developed on the basis of a comprehensive literature review to ensure relevance to the study’s aims. A pilot study was conducted with a small sample of participants who met the inclusion criteria but were not included in the sample size of the main study. The aim of the pilot study was to test the clarity, relevance and completeness of the questions. Feedback from the pilot study participants led to refining the interview guide to improve clarity and ensure that the questions contained the required information. The final interview guide used in the main study can be found in Suppl. 1. The interview began by asking questions about the patient triage process in the ED. Follow-up questions aimed to clarify the concept of caring, patient safety and the factors that affect caring and patient safety in the triage process. The questions were not fixed during the interviews, but the emerging concepts and categories guided the interviews during the study. Data collection was completed on data saturation when the ideas were repeated, and no new data that aligned with GT were identified [30]. The final interview was undertaken with the 19th participant.

Theoretical sensitivity assisted in achieving saturation. Eight years of clinical experience in the ED helped the first author strengthen his theoretical sensitivity, contributing to his ability to listen attentively and respect both the participants and the data they provided [30]. Field notes were taken during the interviews, which raised the abstraction and conceptualisation of the data in advancing the emerging theory [36]. Interviews were transcribed verbatim; omissions in speech, repetitions of words, missed syllables, and single pronouns were omitted. We adhered to COnsolidated criteria for REporting Qualitative research (COREQ) Checklist [37].

### Data analysis

Data analysis was conducted through four coding phases according to the GT methodology [30, 38]. Open, axial and selective coding and a final conditional matrix were used, representing the hierarchical, systematic approach recommended by Corbin and Strauss [30] and involving a constant comparative method with iterative data collection and analysis [30]. In the first step, framed open coding, the all researchers performed a line-by-line review of each transcript, underlining and highlighting lines of interest. A list of similar codes was grouped into subcategories, and data that did not match were set aside for further examination. Axial coding involves grouping categories, reanalysing them as potential concepts, and combining concepts [39]. The paradigm analysis tool and its three characteristics (conditions, actions-interactions and consequences) helped to develop categories and their relationships and to identify the phenomenon or main category of research [30]. In the final step, selective coding was performed to integrate all emerging categories into one category as the central phenomenon, defined as the core category. Finally, all categories and the core category have developed a substantive theory by the conditional matrix [38]. MAXQDA Analytics Pro 2022 was used to support data management.

### Transparency and trustworthiness

To ensure transparency in reporting our findings, we used the COREQ guidelines [37], and for trustworthiness, we followed the Lincoln and Guba [40] criteria for qualitative research. Furthermore, the use of GT and adherence to its guidelines have increased the credibility of our study. The authors had previous experience with qualitative research, which increased the reliability of the study. Participants’ validation ensured audibility. Participants were asked to review the transcripts of their interviews and provide feedback. Additionally, they were invited to confirm or reject the researcher’s interpretations of the data. This step ensured that their views were accurately reflected and enabled any necessary corrections to be made. This constant checking back with participants confirmed that the theory was grounded in the data. Ongoing collation of data, memos and field notes helped this process of reviewing and guiding questions until all categories were theoretically saturated and there was no further scope for exploring the phenomena. All researchers carefully reviewed the data and participated in the entire analysis process. Credibility was achieved by synthesising the categories most representative of the data. The authors discussed emerging themes and findings and reached a consensus to ensure the reliability and validity of the results. Transferability was ensured through semi-structured interviews conducted by updating interviews based on interviews. To ensure content validity, a combination of purposive and theoretical sampling allowed participants to speak freely during the interviews and share detailed descriptions of their experiences. This article describes in detail the various phases of the study and presents the findings with relevant quotations to help convey the results (Dependability) [40]. To achieve trustworthiness, we also analysed the data using the hierarchical, systematic approach recommended by Corbin and Strauss [30], which included the process of theoretical sampling combined with the constant comparison method.

### Ethical considerations

The National Medical Ethics Committee approved the study design, data collection tools and consent forms (Ref. no.: 0120–558/2017/14). Before beginning the study, the researcher introduced himself to the participants, explained the study objectives and methodology, obtained their written informed consent, and assured them that personal data would be treated as strictly confidential. Moreover, they were informed that they have the right to withdraw at any stage. Confidentiality and anonymity were ensured by removing all identifying information before processing the data.

## Results

Of 19 TNs, 15 were female, and four were male. The mean age was 33.79 years and ranged from 24 to 56 years, and their work experience in triage ranged from one year to six years.

**Fig. 1:**
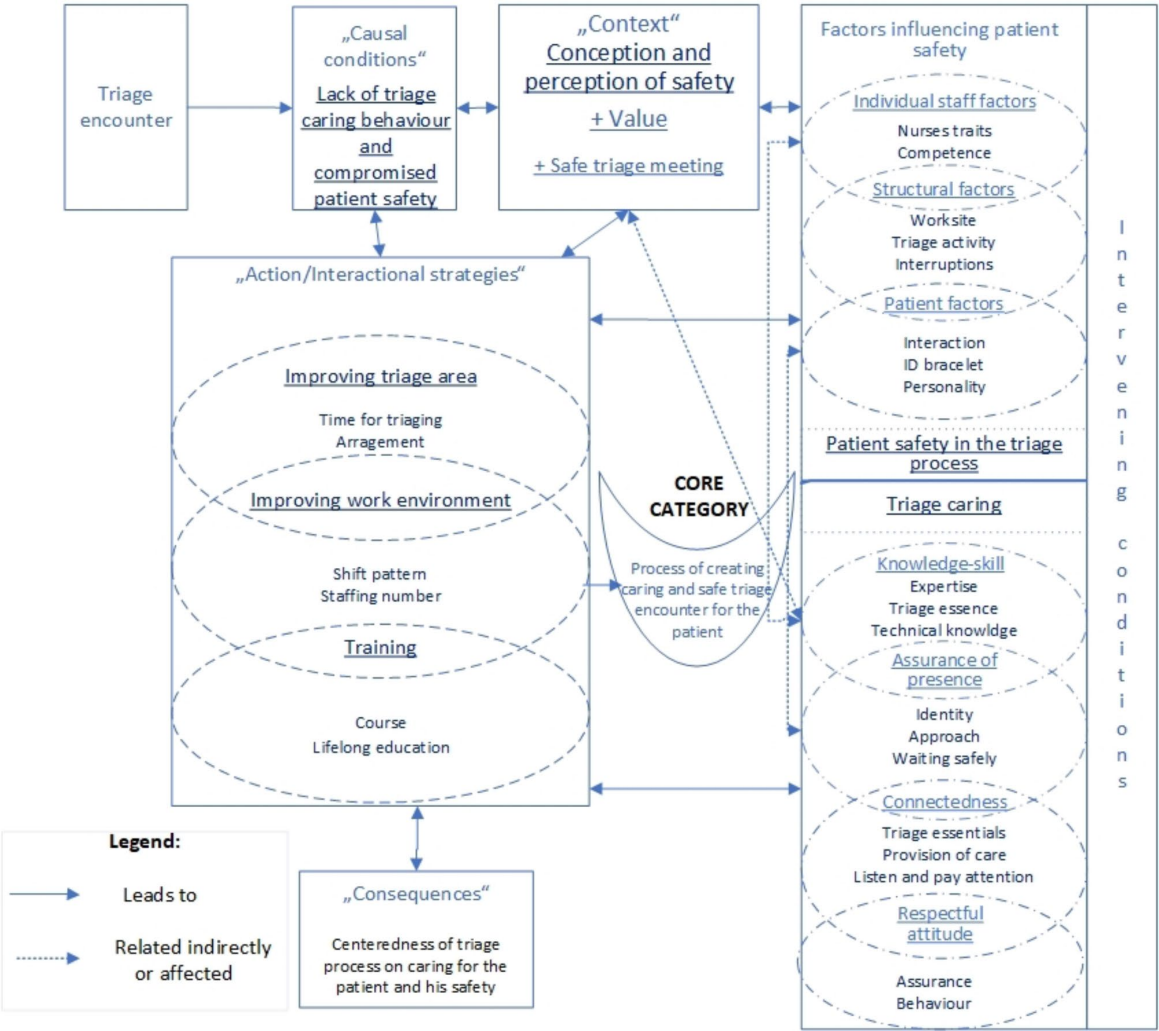
A model of the patient caring-safety encounter in the triage process

The analysis of the interviews generated a core category: “Process of creating caring and safe triage encounter for the patient” and two main categories explaining the Process of caring behavior and patient safety during triage encounter. The central phenomenon is the balance between: (1) Triage caring; and (2) Patient safety in the triage process. A substantive model has been developed to represent the theory and its interrelated processes from the moment the triage caring and safe encounter between the patient and the TN begins (Fig. 1). Each category is presented separately; verbatim quotes attributed to individual participants in the sample are included to support the coding and to illustrate the GT.

### Process of creating caring and safe triage encounter for the patient

The results show that caring for the patient is the basis for ensuring safety. According to the TN, only through careful observation, foresight and work experience can TN improve patient safety. TN highlighted that where caring behavior is present, there is also greater patient safety. Patient safety may be compromised if the TN does not show caring behavior towards the patient.

Through the analysis, we developed the two main categories that explain the process of creating caring behavior and patient safety during triage encounter: (1) Triage caring; and (2) Patient safety in the triage process.

### Triage caring

Within the category “Triage caring”, four subcategories were developed: (1) Assurance of triage nurses’ presence; (2) Connectedness; (3) Respectful attitude; and (4) Knowledge and skills.

#### Assurance of triage nurses’ presence

The assured presence of a TN at the triage workplace represents one of the most basic needs of patients. It includes listening to and focusing on the patient, verifying their identity and ensuring a safe wait for treatment. First, they must listen to the patient, not interrupt them during the conversation and pay attention to them.

*“The onus is on us nurses to listen really well to the patient…” (TN15)*

TN stressed the importance of continuously checking the patient’s identity. They constantly check their data and, for this purpose, attach an identification bracelet to every patient under their care:

*“All patients, regardless of triage category, get a bracelet on arrival to ensure their safety and prevent further mistakes under our care.” (TN7)*

After assessing the patient’s health status and assigning the triage category, participants emphasised the importance of regular monitoring of the patient in the waiting room. This includes continuous monitoring of the patient’s health status in the waiting area to ensure their safety while they wait.

#### Connectedness

Every patient who walks into the ED is a concern for the TN as they are the first person to encounter the patient. For them, caring for the patient is the mission of their profession and the greatest thing in doing their job. Caring for TN means ensuring continuous and timely patient care according to their acute medical condition and is considered crucial for their subsequent treatment:

*“Caring for the patient means me the mission of my profession, which includes the responsibility and duty to ensure quality and safe treatment according to the patient’s level of acuity.” (TN1)*

*“I think of caring for the patient in the way that when performing my work, as a triage nurse, I not only perform triage but also “take care” of the patient in a broader sense.” (TN14)*

The cornerstone of triage in the context of the connection with the patient for TN are conversation, understanding, trust, explanation and health education. Patients are often frightened and worried about their own health, so it is essential to talk to them and explain the course of their treatment.

*“We try to listen to the patient why he is coming, not to interrupt him, let him tell us what his problem is, and then we advise him or tell him what is better to do or, yes, how his process will be during this triage or waiting.” (TN13)*

#### Respectful attitude

TNs also spoke about ensuring a respectful and dignified attitude and an appropriate environment during the triage process. TNs highlighted the importance of a professional and polite attitude towards patients.

*“Respectful and courteous attitude towards patients. You take the patient seriously enough not to underestimate him, not to privilege him, to triage him appropriately.” (TN2)*

It is important not to underestimate the patient’s problems and treat each individually. The patient should be invited into the triage room, made comfortable and reassured comfortable feelings, and feel that TN are there for them during the triage.

TN further spoke about ensuring confidentiality between the TN and the patient. The patient needs to feel that they can trust TN and are understood. In this way, the patients state the reason for the arrival and will not omit or withhold important information about his health condition. During this time, privacy must be ensured by closing the triage room doors to establish privacy, intimacy and anonymity.

*“The environment itself has a major impact, as every patient can feel that they can trust the healthcare staff and that their treatment will be safe and of high quality.” (TN7)*

#### Knowledge-skills

Performing patient care during patient triage also requires knowledge and skills in professional and technical expertise. To handle the triage process with care, the TN must have broad and specialised knowledge in patient care, emergency medicine and triage system:

*“Patient care for me means that I, as a triage nurse, can triage the patient correctly with my knowledge and competence.” (TN9)*

Careful triage nursing involves questioning, observing, recognising, assessing, triaging and transferring the patient to the right workplace. TN emphasise that a TN must be able to ask the right question and identify the patient’s main problem. As well as asking questions, observation is also important, as the patient’s visual appearance is also important.

*“To me, as a triage nurse, taking care of the patient means the best, that I know how to assign him based on some correctly asked questions in the appropriate first in the appropriate triage algorithm.” (TN2)*

### Safety in the triage process

Within the category Safety in the triage process, three subcategories were identified: (1) Conception and perception of safety; (2) Factors influencing patient safety; and (3) Improving the triage safety.

#### Conception and perception of safety

TN defined triage as a pillar of the ED, a key to an ED, and a vital patient entry point. Furthermore, it presents a daily challenge for them and other healthcare staff. The TNs describe safe triage as the timely and correct patient allocation at the right place and in the appropriate triage category. The TN has to recognise the patient’s urgency, extract the main reason for their arrival at the ED and triage correctly:

*“Patient safety is a sacred thing for me because I am aware that when a patient comes to triage and if I treat him as a triage nurse, I know that I am responsible for him.” (TN15)*

*“Safe triage in the emergency department means that the patient will be triaged into a triage category and,….” (TN7)*

#### Factors influencing patient safety

The identified factors were individual staff factors, structural factors and patient factors.

Among the individual staff factors influencing patient safety, TN exposed their traits, characteristics and attributes, such as intuition and practical and clinical skills. They stressed that learning needs to be reinforced by experience, as patients are often not asked the right questions to obtain a realistic assessment of their acuity due to a lack of knowledge:

*“Well, because here it seems to me that the more triages you do, the more situations you experience, the more cases in your life turns around, the more you pay attention to certain things.” (TN5)*

Within the structural factors, we identified the following factors: worksite, workspace, triage activity and interruptions.

Triage represents the workplace where the assessment of patients’ acuity starts. Therefore, sufficient TN must be deployed at the work site to ensure safety. They further stressed the need for more TNs to relieve the burden on existing TNs:

*“We often don’t have enough staff, and it becomes really challenging. We all know what times are like, with staff shortages and more patients per employee. If we had more triage nurses, it would definitely help to manage the workload better and ensure we can provide safe patient care …” (TN8)*

There is a greater sense of safety for the TN during the triage process if an older and experienced TN is present to advise, assist and support the TN in learning new TNs. Therefore, they stress that beginners in triage should have a supportive mentor by their side. According to TN, triage was not the easiest way to get started - they did a course and then started to work independently.

*“Also, the longer they are there and triage, the more they help you, but not the more you can turn to someone when you are divided, and debate together, and then find a solution”. (TN19)*

For TN, crowding, rush, or a large patient turnover is the biggest reason for compromised patient safety and poor triage quality. TNs point out that this happens to them on a daily basis, mainly due to the shortcomings of the primary healthcare system. According to TN, crowding reduces the triage time because they want to triage the patient as quickly as possible or too quickly, which makes them nervous and under pressure.

*“Too many patients come to the emergency department because of the shortcomings of the primary healthcare system.” (TN7)*

Triage is often interrupted by various factors such as the arrival of paramedics, ringing phones, and knocking of patients and their relatives and colleagues.

*“Then it’s the paramedics who hand someone over to you, claiming that they’re, says, more at risk, or they’d like to hand over the story to you so they can go.” (TN5)*

*“We are also distracted by telephones, which constantly interrupt the work process…” (TN16)*

The physical environment and equipment were often highlighted in the workplace as patient safety factors in the triage process. For safe triage, a sufficient room size with wide doors is essential, which is comfortable for the patient and the TN, equipped with a bed with safety bed rails. They emphasise that the devices must be adequate, functional, calibrated, reliable and regularly inspected:

*“It seems to me that the room must be comfortable, for the patient and the triage person, because then they both feel comfortable in that room and can do their job.” (TN5)*

The last and most important factor affecting patient safety in the triage process, TNs, mentioned patients. They mainly highlighted their interaction with patients, their personality characteristics, and the importance of fitting the patient’s identity bracelet. During triage, situations often arise when the patient gives different information about the reason for coming to the ED than what patients tell the physician later during the treatment:

*“… yes, this is a problem, but then he tells you something and tells the doctor something completely different.” (TN2)*

#### Improving the triage safety

Improving triage safety as the subcategory consisted of three elements: triage area, work environment and training. To improve patient safety, TNs first highlighted the appropriate arrangement of the triage area in terms of the expansion and arrangement of this space. They also want more time for triage to identify emergencies and therefore provide better quality and safe care for the patient in the triage area:

*“And, of course, spatially, I think it would still have to be sorted out a bit. That seems to me to be the point.” (TN5)*

Almost all TN spoke of needing additional trained and experienced TNs to relieve their workload. TN stated that the schedule and length of triage work should also change:

*“The safety of patients during treatment would be higher if there were a sufficient number of daily TN.” (TN14)*

Triage training gave them the basics of what triage should look like but not enough knowledge to triage confidently and safely by themself. They missed more practical work under supervision, which would have given them additional knowledge.

## Discussion

GT from the presented study describes TNs’ perceptions of caring behaviors and ensuring patient safety in the triage area. The core category, which connects all categories and enables a comprehensive understanding of the data, was the Process of creating caring and safe triage encounter for the patient. The TNs’ perceptions about caring for the patient and his safety in the triage area show that patient care and safety are inseparably linked and coincide when triaging a patient. Namely, caring for the patient means ensuring the patient’s safety at the same time. Moreover, applying caring behavior during triage encounter results in greater patient safety.

Emergency nursing care begins with triage, which aims to support the healthcare staff in identifying patients needing immediate medical intervention [2, 28, 41]. According to Johnson, Punches and Smith [14], the triage process impacts safety because TNs must identify and recognise who is life-threatened and needs immediate help. We found that patient safety is very important to TN, as they believe their wrong decision can seriously affect the patient’s health or life. For TNs, patient safety means the identification and recognition of an emergency, the correct classification of the patient into the appropriate category according to the identified problems, and the correct referral of the patient to the proper ward in the shortest possible time. The results of our study are in concordance with the findings of the study by Varndell, Hodge [4], who reported that the primary purpose of triage is to ensure that the level of emergency triage care provided is commensurate with clinical urgency. During the triage process, nurses in our research highlighted important factors contributing to patient safety, such as individual staff, structural and patient factors. Our results aligned with the study by Varndell, Hodge [4] that reported that patient safety relies upon the staff traits such as experience, education and training of triage nurses. Experience and knowledge are grouped together because no course gives you enough knowledge for such a demanding job. According to Reblora, Lopez [3], experience often contributes to understanding patients’ conditions and asking the right questions to determine the severity of health problems. Staffing shortages in complex and unpredictable working conditions, such as a triage area, present a challenge for nurse managers in organising work to provide the best, safest, and quality patient care [28]. Participants indicated that the schedule and duration of triage work affected their concentration and caused fatigue, which they felt could lead to urgent situations being overlooked and patient safety being jeopardised. These perceptions are supported by the findings of Reay, Smith-MacDonald [17] who reported that too many consecutive shifts or long shifts in triage can negatively affect patient care decisions and interactions. In addition, participants noted that weekend shifts in triage are perceived as particularly long and stressful, especially when they last 12 h.

Caring for the patient is fundamental [42] in the ED for quality, safe and holistic care for emergency patients [43]. Our results regarding caring for the patient in the triage process show the importance of knowledge, respectful attitude, connectedness and ensuring the presence of the TN. TN stressed that caring for the patient in the triage process means holistic, quality and safe treatment; at the same time, it is the profession’s mission for them. The connectedness-related behaviors include explaining, giving health education advice, spending time with patients, being tireless toward patients, and involving patients in their triage care plan. TNs report that the care is often compromised due to overcrowding and staff shortage because patient safety is prioritised in these cases. Consequently, when caring behavior in the triage is evident, patients feel comfortable, safe and trusting of the TNs who care for them [2]. Therefore, the TN approach is fundamental in the triage encounter. According to Holopainen, Nyström and Kasén [24], a caring triage encounter occurs between two equals, one of whom is a nurse and the other a patient. Nursing presence in the triage process occurs when the triage nurse interacts with the patient, listens to the patient’s story, actively listens to the patient, establishes eye contact and empathy and uses a calm speech. However, the presence occurs through nurses being with wholeness at the patient along with performing the triage nursing care procedures [44]. In a caring triage encounter, a triage nurse is perceived as caring, competent, concerned and respectful of the patient as a person [24]. The TN invites the patient to the triage room, which should be comfortable and reassuring, and in which the patient feels at ease. TN further emphasises that the relationship between the patient and TN must be respectful, polite, and non-underestimating, and the patient must feel that the TN are available. The patient-triage-nurse relationship is jeopardised in the event of a breach of patient confidentiality [2]. TN spoke about establishing a confidential relationship, where the patient confides the main problem or reason for coming to the emergency department. Therefore, the triage room doors must be closed to ensure patient privacy. According to Koskimies, Koskinen [45], patients feel uncomfortable when confidentiality and privacy are breached, making sharing important information about their medical condition difficult. TN describe that during triage, patients often withhold certain information or tell different information about their health condition than they later tell the physician. Hence, if the TNs do not obtain a complete picture of the patient’s health condition due to withheld medical information, the patient is consequently deprived of optimal care in the ED, or, as a result, the patient’s life is even endangered [2]. To prevent a patient from becoming endangered, the caring triage encounter must establish a trusting environment, where, according to Holopainen, Nyström and Kasén [24], the patient can express their feelings and emotions, and TNs are open and attentive with an ability to understand the patient’s needs and problems. Therefore, it is necessary to point out that patient care in ED starts with triage, which is crucial for the patient’s subsequent treatment. If care is not present in the triage process, the TN can quickly overlook the seriousness of the patient’s condition.

Overall, caring for the patient and the patient’s safety in the triage process are inseparably linked with identifying and recognising patient severity, patient ID verification, sorting patients in the right triage category, asking questions, talking with the patient, handover and TN knowledge. In addition, establishing a caring environment where patients are placed at the centre of care aims to make the patient feel calm and confirmed. As a result, this leads to the provision of accurate information about the reason for arrival to the ED and accurate triage, which is essential for patient safety. TN should use interaction that invites and encourages each patient to participate and negotiate in decision-making about their care. This interaction is a core component of caring science and high-quality nursing care, facilitating patient safety [28].

### Limitations

The study has limitations that should be considered when interpreting its findings. This study provides a detailed in-depth analysis of a challenging professional practice area. The study was conducted during the COVID-19 restrictions, which may have influenced the stories and experiences of the participants. The GT stresses processes and meanings of the phenomenon in the local context; therefore, findings cannot be generalisable to all EDs. However, recruitment was limited to TNs employed within one ED. The limitation of our study is that a wider group of triage nurses were not directly involved in the planning phase. Whilst the study was informed by our professional experiences as triage nurses. In addition, the perspectives of the public were not included in the study design. Future research should incorporate these viewpoints to ensure that the research better meets the needs and concerns of both healthcare professionals and the public. Purposive sampling was used to select triage nursing participants, minimising biases by considering factors like gender, age, education, and professional experience to ensure diverse perspectives and minimise homogeneity. The participants’ context in terms of their experiences and demographics may have influenced the way in which they expressed and discussed their views and attitudes. Finally, one of the limitations of our study is the consideration of the possible influence of cultural and regional factors that could affect the perception and implementation of caring behaviours among triage nurses. Cultural norms and regional workplace dynamics may significantly shape the way caring and safety behaviours are enacted and experienced. These contextual differences could affect the generalisability of our findings. Nevertheless, our study incorporates a valuable view of experiences TN on this matter. Saturation was achieved with the data provided by the participants, but different insights could be obtained from TNs who did not participate in the study. All TNs perform triage according to the same guidelines and triage system and have a standardised computer programme familiar to all EDs in the country. Including participants from other health care and triage systems using different protocols could strengthen this study. Finally, the patients as triage stakeholders were not included in the study. The patient’s experience during the triage process in terms of care and safety assurance can give additional valuable information to improve the triage process. Therefore, further research on this topic is recommended.

## Conclusions

The study underlines the inseparable link between caring behaviors and patient safety during triage in EDs. The participants believe that effective caring directly enhances patient safety, which emphasises the need for caring and safe triage. TNs perceive that caring and safety are inseparably linked in the triage process, as caring for the patient ensures their safety simultaneously. Triage ensures that the ED’s quality and caring behavior are appropriate and safe for the severity of the patient’s illness acuity. TNs experience and perceive caring for the patient and their safety through a respectful attitude, connection with the patient, ensuring presence, conscious focus on patient needs and problems, safe triage encounter and value of safety. Factors affecting safety include TNs’ characteristics and employment patterns, workplace factors and related interruptions. TNs are at the forefront in the dynamic environment of ED and carry the burden of providing care and safe triage that patients recognise as caring. The triage process must put each patient at the centre of triage care, treating them as individuals rather than as present health conditions in which it is.

### Implications for healthcare management

Our findings have significant implications for healthcare management and clinical practice. This GT identified two important elements that impact the current triage process: caring for the patient and the patient’s safety in the triage process. The findings of this study can create awareness of the importance of caring for the patient and their safety in light of a heavy ED workload. TNs are split between providing care and responding to patient care needs while ensuring patient safety. Healthcare organisations must provide a work environment where TNs can express and experience all dimensions of caring while ensuring that the triage environment is safe for patients. Special care is also needed when planning the work schedule for TN, where shift patterns, lengths, and the necessary rotation within a shift must be considered to prevent fatigue and errors in the triage process.

### Electronic supplementary material

Below is the link to the electronic supplementary material.

Supplementary Material 1

## Data Availability

The datasets obtained because of the study are not available for sharing due to the need to protect the anonymity and confidentiality of the participants, as well as to respect their sensitive contributions. Although the corresponding author possesses the data, it was generated, utilised and analysed specifically for the present study. However, interested parties may obtain the data from the corresponding author upon making a reasonable request that aligns with the informed consent forms signed by the participants.
