# Supplementary material for: Emergency triage nurses’ perceptions of caring behaviors and the safety of the patient during triage encounters: a grounded theory study
Source: BMC Nurs. 2024 Jul 3;23:453. doi: 10.1186/s12912-024-02122-5 (PMC11221186; doi:10.1186/s12912-024-02122-5)
Supplement: Supplementary file 1 — Supplementary Material 1 [file 12912_2024_2122_MOESM1_ESM.docx]

# Suppl. 1: Overview of interview questions

1. Please describe how a triage encounter is conducted in the emergency department.

2 What does caring for a patient in the triage mean to you?

3 What do you imagine under the caring of a patient?

4 Where is this caring evident in the triage process, and what factors influence it?

5 How would you describe patient safety in the triage process?

6. What does patient safety mean to you?

7 What contributes to patient safety during the triage process besides triage systems?

8 In your opinion or experience, what are the main reasons for compromised patient safety during triage?

9 Why do you think caring for a patient is important for patient safety?
